# The Role of Nutrition in Chronic Disease

**DOI:** 10.3390/nu15030664

**Published:** 2023-01-28

**Authors:** Sareen S. Gropper

**Affiliations:** Christine E. Lynn College of Nursing, Florida Atlantic University, Boca Raton, FL 33431, USA; sgropper@fau.edu

According to the Centers for Disease Control and Prevention, six out of every ten adults in the United States have at least one chronic disease, and about four in ten have two or more chronic diseases [1]. Chronic diseases, i.e., conditions that occur for at least one or more years and necessitate ongoing medical care, include diseases such as cardiovascular conditions, cancers, diabetes mellitus, and Alzheimer’s disease. These conditions are also among the leading causes of death globally, accounting for 70% of all deaths around the world [2,3,4].

Diet, often considered as a lifestyle factor, contributes to the development of many chronic conditions including obesity, cardiovascular disease, hypertension, stroke, type 2 diabetes, metabolic syndrome, some cancers, and perhaps some neurological diseases. Moreover, one medical condition, when present, often contributes to the development of other medical conditions, such as the impact of obesity or excess body weight/fat as a risk factor for conditions including type 2 diabetes, hypertension, metabolic syndrome, and some cancers, among others. This Special Issue features research conducted by Ding and coworkers [5], which demonstrated significant associations between weight change during the different phases of adulthood and the risk of non-alcoholic fatty liver disease. The authors reported that their findings, if causal, could translate to the prevention of about 73% of incident non-alcoholic fatty liver disease if individuals maintained a healthy body mass index across adulthood [5].

Studies employing modifications of “usual” dietary practices are sometimes used as a means of evaluating diet’s impact on disease risk and/or on specific disease risk factors. Two papers in this Special Issue focus on the impact of diet on chronic disease risk. First, the study by Kim and Giovannucci [6] examined the long-term impact of plant-based diets and disease risk in an Asian population. Their findings indicated that healthier plant-based diets are associated with a lower incidence of hypertension and type-2 diabetes, especially among those with a family history of the disease [6]. Moreover, in this Special Issue, under the area of diet and disease risk, is a systematic review written by Giosue and colleagues [7]. These researchers evaluated published studies that examine dairy product consumption and disease risk with a focus on cardiovascular disease, while also addressing its association with factors that contribute to disease risks such as body weight, fasting blood glucose, glycated hemoglobin, blood pressure, and inflammation to name a few. Their findings may indeed contribute to changes in dietary guidelines regarding dairy product consumption [7].

Dietary guidelines in many countries have more recently focused on overall dietary patterns versus individual nutrient intake and disease risk. Yet, it is well established that nutrient deficiencies or suboptimal nutritional status can contribute to the development of diseases and/or health problems (for example inadequate vitamin D and/or calcium status and their impact on bone development/maintenance, as well as inadequate vitamin K status and its impact on blood coagulation, among other things). The study by Li and colleagues [8] evaluated serum 25-hydroxyvitamin D concentrations in adults who had previously experienced a stroke to assess the vitamin’s associations with risk for a recurrent stoke. A J-shaped relationship was found between serum 25-hydroxyvitamin D concentrations and risk of recurrent stroke in these adults with a stroke history, and as the author’s point out, there is a clear need for further studies to examine if there is a possible cause–effect relationship [8]. Just as impairments in vitamin D status can negatively impact critical body functions and be negatively associated with disease risk, other nutrients play a multitude of other roles in the intricate workings of the cells of the body. Das [9] explored the roles and mechanisms of the fatty acid arachidonic acid as a mechanotransducer of renin cell baroreceptor.

While dietary modifications can help to prevent the development of many chronic diseases, once a condition has developed, changes to a person’s usual diet are often needed to assist with disease (or symptom) management. The article by Dynka, Kowalcze, and Paziewska [10] reviewed the effectiveness of ketogenic diets in the management of epilepsy and other neurological conditions, as well as the diet’s possible action mechanisms.

In addition to the role of dietary modifications in the treatment of disease or its symptoms, disease management may also include the use of dietary supplements. The effectiveness of oral nutritional supplements for individuals with diabetes/prediabetes was examined in two studies in this Special Issue. In the paper by López-Gómez and colleagues [11], the researchers demonstrated the effectiveness of a diabetes-specific oral supplement on reducing the prevalence of malnutrition and sarcopenia in patients with diabetes/prediabetes. Similarly, the effectiveness of nutrition supplementation and education (versus standard care for wound treatment) on inflammatory biomarkers was assessed in patients with diabetic foot ulcers by Basiri and coworkers [12]. Supplementation and education were found to positively control inflammation in the patients.

While an array of oral nutritional products on the market provides nutritional support to individuals with chronic diseases, investigations into the effectiveness of vast numbers of nutraceuticals targeting disease prevention and treatment are also exploding in the scientific literature. One such condition is age-related macular degeneration, as explored by Luján and colleagues [13]. The paper provides a detailed examination of the role of mitochondrial dysfunction in the progression of the disease, as well as an examination of the nutraceuticals/drugs that may be able to up-regulate mitophagy and mitochondrial biogenesis to enable the possible prevention or control of the disease. Some of the nutraceuticals/drugs that are examined include ferulic acid, melatonin, urolithin A and glucosamine, metformin and berberine, lipoic acid and broccoli sprout extract, and fibrate drugs and astaxanthin. Moreover, the effectiveness of extra-virgin olive oil as a means of reducing gut-permeability-derived low-grade endotoxemia in adults with impaired fasting glucose is presented in a study by Bartimoccia and colleagues [14].

Finally, the role of nutritional support for chronic disease is not complete without the inclusion of some studies investigating the complex relationships of diet/food and of disease with the gut microbiota. Zhang and colleagues [15] studied the associations between habitual diet patterns and gut microbiota in Chinese adults. The authors found that the intake of specific foods or food groups, such as whole grains, vegetables, and red meats, among others, was associated with changes in the abundance of specific genera and species of gut microbiota. Moreover, the review by Araujo, Borges-Canha, and Pimentel-Nunes [16] explored differences in the gut microbiome among individuals with metabolic syndrome (versus healthy adults) as well as the potential for probiotics and/or synbiotics to modulate the microbiome in an effort to mitigate some of the metabolic disturbances in some individuals with metabolic syndromes.

The studies featured in this Special Issue, as well as in the wider scientific literature at large, are critical in furthering the knowledge of diet and nutrition support of chronic disease. As biological mechanisms underlying chronic diseases continue to be elucidated and the causes and consequences of diet-related conditions are better characterized, new intervention strategies can be implemented, studied, and evaluated. These findings will not only assist in the formation of additional evidence-based dietary guidelines, but may also help healthcare professionals educate their patients and facilitate their adoption of healthful eating behaviors [17].
